# ARG1-polyamine axis: cell-type-specific functions in disease pathogenesis and therapeutic targeting

**DOI:** 10.3389/fimmu.2026.1744890

**Published:** 2026-03-19

**Authors:** Lexing Li, Guoyan Zhu, Mengdie Chen, Bingqing Qiu, Yujia Li, Shiyu Liu, Wei Gu, Leilei Liu

**Affiliations:** 1 College of Life Science and Technology, Wuhan University of Bioengineering, Wuhan, Hubei, China; 2 National Key Laboratory of Agricultural Microbiology, Wuhan, Hubei, China; 3 Department of Biochemistry and Molecular Biology, School of Laboratory Medicine, and Anhui Provincial Key Laboratory of Tumor Evolution and Intelligent Diagnosis and Treatment, Bengbu Medical University, Bengbu, Anhui, China

**Keywords:** ARG1, immune regulation, metabolic reprogramming, polyamine metabolism, precision therapy

## Abstract

ARG1 catalyzes the conversion of L-arginine to L-ornithine, urea, polyamines, and L-proline, thereby balancing nitrogen detoxification with tissue-specific roles in proliferation and immunity. This review delineates the context-dependent functions of ARG1 across diverse cell types—including tumor cells, immune cells, endothelial cells, keratinocytes, and stem cells. In tumors, ARG1 drives immunosuppression and metabolic reprogramming but can paradoxically suppress tumorigenesis. Immune modulation via ARG1-polyamine crosstalk regulates T cell differentiation, macrophage polarization, and microbiota interactions, influencing infection and autoimmunity. Endothelial ARG1 exacerbates obesity-related vascular dysfunction, while keratinocyte ARG1 impacts wound healing and psoriasis. Emerging therapies—such as ARG1 inhibitors, engineered extracellular vesicles, and microbiome interventions—show preclinical promise in cancer, cardiovascular, and neurodegenerative diseases. By mapping ARG1’s spatiotemporal metabolic networks, this work highlights its dual roles and positions ARG1 as a central player for precision medicine in complex pathologies.

## 1 Introduction

**Figure 1:**
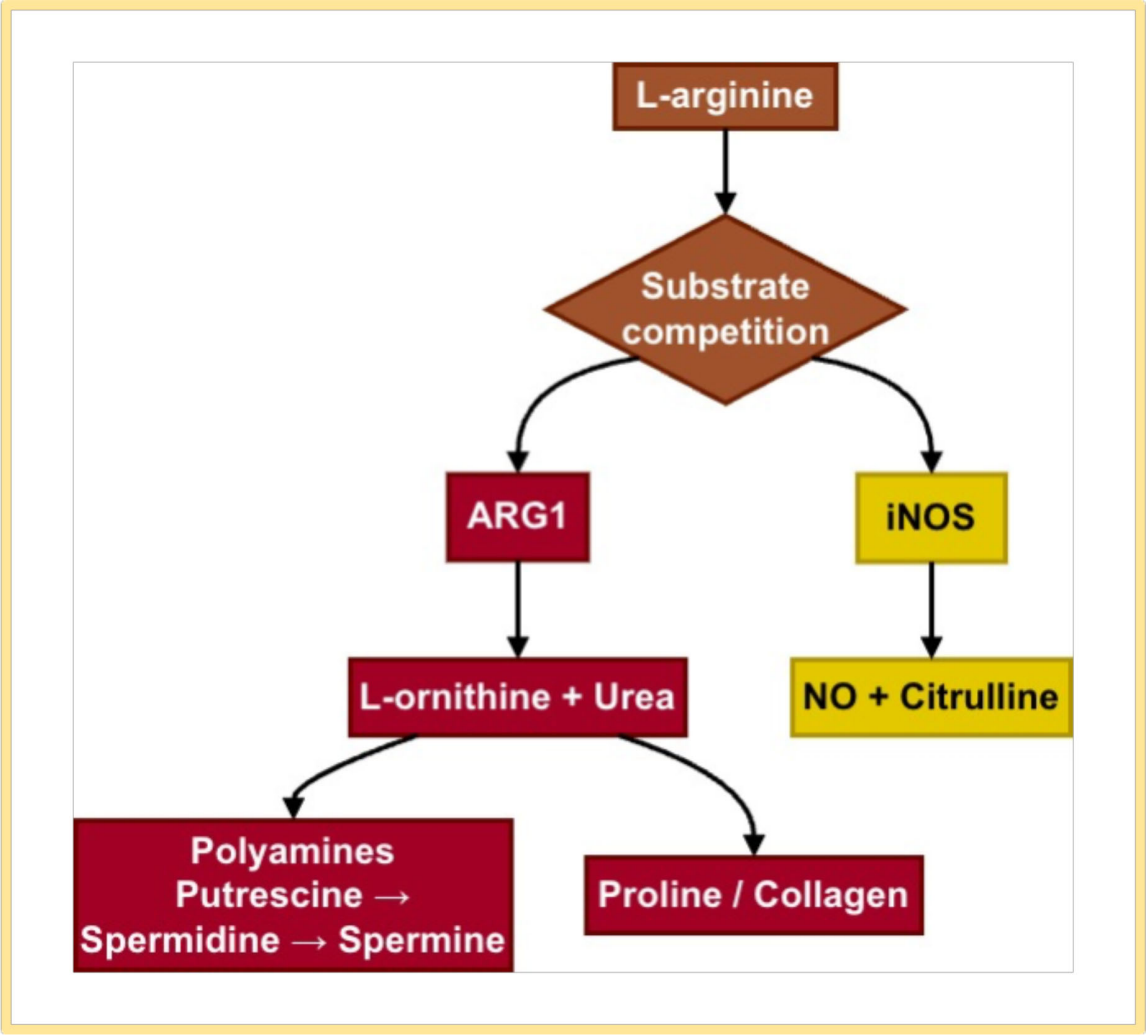
ARG1 and iNOS compete for L-arginine as a common substrate, leading to divergent metabolic and functional outcomes. ARG1 pathway: ARG1 hydrolyzes L-arginine to L-ornithine and urea. L-ornithine serves as a precursor for polyamine synthesis (via ODC1) and L-proline/collagen production (via OAT), supporting cell proliferation, matrix remodeling, and immunosuppression in various disease contexts. iNOS pathway: iNOS oxidizes L-arginine to nitric oxide (NO) and citrulline. NO mediates antimicrobial responses (via iNOS), while also playing key roles in vasodilation (primarily via eNOS) and neurotransmission (via nNOS). Its reduction due to ARG1 upregulation contributes to endothelial dysfunction and immune dysregulation. This competitive interaction underscores the dual role of L-arginine metabolism in health and disease, influencing immune response, vascular function, and tissue repair.

Urea cycle (UC) is essential for nitrogen and ammonia detoxification, with ARG1 (arginase 1) playing a key role in converting L-arginine to L-ornithine and urea (1). L-arginine is a semi-essential amino acid, a substrate for nitric oxide (NO) production by nitric oxide synthase (NOS), and a precursor to a variety of metabolites including ornithine, creatine, polyamines and guanidine. ARG1 competes with NOS for substrate L-arginine to produce ornithine and urea (2). This competitive metabolism of L-arginine initiates two critical and functionally opposing pathways, fundamentally shaping NO bioavailability, polyamine synthesis, and ultimately, cellular fate in health and disease (**Figure 1**). The upregulation of ARG1 gene expression and activity enhances the conversion of L-arginine to L-ornithine and urea. L-ornithine is the main precursor to the production of polyamines and L-proline. Ornithine decarboxylase (ODC) catalyzes the conversion of L-ornithine to putrescine, the first and rate-limiting step in polyamine synthesis (3). Ornithine aminotransferase (OAT) converts L-ornithine to pyrroline-5-carboxylate (P5C), a key precursor for L-proline and collagen production (4). Polyamines and L-proline are essential for cell proliferation and collagen synthesis, respectively (5). While ARG1 is widely recognized for its immunosuppressive role via L-arginine depletion, its enzymatic product L-ornithine serves as a precursor for polyamine synthesis, which is critical for cell proliferation, differentiation, and immune modulation. Emerging evidence suggests that ARG1-driven polyamine production contributes to disease pathogenesis in a cell-type-specific manner, influencing processes such as tumor progression, fibrosis, and chronic inflammation (6, 7). Thus, the “ARG1-polyamine axis” represents a key metabolic and signaling hub that integrates immune and tissue remodeling responses across various diseases. However, in different tissues or cells, ARG1-polyamine metabolism exhibits different functions. L-arginine serves as a common substrate for ARG1 and NOS, but the two have opposite effects on vascular remodeling (8). Therefore, this paper summarizes the gene expression and functional specificity of ARG1-polyamine metabolism in diverse cell types—including tumor cells, tumor-associated cells, immune cells, endothelial cells, keratinocytes, smooth muscle cells, and stem cells—as well as the interactions between macrophages and other cells, and between polyamine metabolism and microorganisms. This comprehensive analysis reveals that the ARG1-polyamine axis operates as a critical metabolic branch point, directing cellular fate through polyamine−dependent proliferation or L−proline/collagen−mediated matrix remodeling, thereby providing a theoretical foundation for precise disease treatment and targeted drug development. Human ARG1 is a binuclear manganese metalloenzyme that catalyzes the conversion of L-arginine to L-ornithine and urea. Its structure facilitates substrate competition with NOS for L-arginine, positioning it as a critical regulator of nitric oxide (NO) bioavailability and polyamine synthesis. Understanding the structure and regulation of ARG1 is therefore fundamental for developing targeted therapeutic strategies (9). Having established the fundamental role of ARG1 in nitrogen metabolism and its broad physiological implications, we now delve into the molecular mechanisms and signaling pathways that precisely regulate its expression and activity.

## 2 Molecular mechanisms and regulatory signaling of ARG1

The expression and activity of ARG1 are tightly controlled by a multi-layered network of signals, ranging from cytokine receptors to epigenetic modifiers, which collectively determine its cell-type-specific functions.

### 2.1 Core cytokine signaling pathways

The STAT6 (signal transducer and activator of transcription 6) pathway is a primary inducer of ARG1, particularly in macrophages polarized by interleukin-4 (IL-4) and IL-13 (10, 11). This canonical signaling can be potentiated by concurrent activation of p38 mitogen-activated protein kinase (MAPK) or AMP-activated protein kinase α (AMPKα) (10, 12). Negative feedback is provided by mechanisms such as VEGFR1 (vascular endothelial growth factor receptor 1) tyrosine kinase activity, which suppresses IL-4-induced ARG1 to prevent excessive M2 polarization (11). In pathological settings such as cancer, alternative pathways emerge. For instance, granulocyte-macrophage colony-stimulating factor (GM-CSF) can induce ARG1 in myeloid cells via STAT3 and p38 MAPK independently of STAT6, a mechanism exploitable for immunotherapy (13).

### 2.2 Transcriptional and post-transcriptional control

Beyond cytokine receptors, direct transcriptional regulators fine-tune ARG1. The transcription factor Fra-1 binds to the ARG1 promoter to repress its expression, exacerbating inflammation in arthritis (14). Conversely, in cancer-associated fibroblasts (CAFs), Discoidin Domain Receptor 2 (DDR2) activates SNAI1 (snail family transcriptional repressor 1) to bind the ARG1 promoter, driving collagen production and metastasis (15). Post-transcriptionally, microRNAs such as miR-135a-5p can inhibit STAT6, thereby reducing IL-4 secretion and subsequent ARG1 expression in tumor-associated macrophages (TAMs) in non-small cell lung cancer (NSCLC) (16).

### 2.3 Epigenetic modulation

Epigenetic mechanisms provide another layer of regulation. DNA methylation status of the ARG1 promoter correlates with its expression, as seen in asthma and in myeloid-derived suppressor cells (MDSCs) where hypomethylation is linked to high ARG1 (17, 18). RNA N6-methyladenosine (m6A) modification also plays a role; ALKBH5 (AlkB homolog 5)-mediated demethylation destabilizes ARG1 mRNA in MDSCs, reducing immunosuppression (19). Histone modifications are equally crucial: histone deacetylase 4 (HDAC4) recruitment to the ARG1 promoter during dendritic cell differentiation facilitates activation (20), while mutations in epigenetic regulators like tet methylcytosine dioxygenase 2 (TET2) and DNA methyltransferase 3 alpha (DNMT3A) in myelodysplastic syndromes are associated with increased ARG1 (21). In hepatocellular carcinoma TAMs, this induction involves succinate-mediated increases in H3K4me3 at the ARG1 promoter, enhancing its transcription (22).

### 2.4 Regulation by metabolites, microenvironment, and exogenous factors

The metabolic and tissue microenvironment significantly influences ARG1. Metabolites like 25-hydroxycholesterol and succinate can act as signaling molecules to induce ARG1 (10, 22). The gut microbiota and its impact on L-arginine metabolism also regulate ARG1 activity, as observed in colitis models (23). Furthermore, pathogens can directly modulate host ARG1; for example, Salmonella infection reprograms L-arginine metabolism in IL-4-stimulated macrophages (24), and the fungal protein inositol polyphosphate 3–4 kinase (IP_3-4_K), a homolog of ARG1 in Cryptococcus neoformans, drives virulence (25). A summary of these diverse regulatory inputs across disease contexts is provided in **Table 1**.

**Table 1:** Induction and regulation of ARG1 across disease contexts.

Disease/context	Key inducers/regulators	Core mechanism/pathway	Reference
Cancer	GM-CSF	STAT3/p38 MAPK signaling in myeloid cells.	(13)
	Metabolite (Succinate)	OXCT1-mediated ketolysis increases H3K4me3 at the ARG1 promoter in TAMs.	(22)
	lncRNA (LVBU)	Stabilizes BCL6 to block p53-mediated repression of urea cycle/polyamine genes (ARG1, OTC, ODC1).	(26)
	Immune Checkpoint (VISTA)	Promotes STAT3-dependent polyamine synthesis in MDSCs.	(27)
	Collagen Receptor (DDR2)	Activates SNAI1 to bind the ARG1 promoter in CAFs.	(15)
Autoimmunity/Inflammation	Transcription Factor (Fra-1)	Directly binds and represses the ARG1 promoter in macrophages.	(14)
	Metabolite (25-Hydroxycholesterol)	Activates AMPKα to enhance STAT6 phosphorylation and ARG1 induction.	(10)
	Phosphatase (PP6) downregulation	Leads to C/EBP-β activation and ARG1 upregulation in keratinocytes.	(7)
Neurodegeneration & Injury	Pathogen/Damage Signals	ARG1 is expressed exclusively by infiltrating myeloid cells, not resident microglia, in CNS injury.	(28)
	Viral Vector (AAV)	Direct overexpression of ARG1 in the CNS.	(29)
Metabolic & Vascular Disease	Hormone (Angiotensin II)	Induces ARG1 expression/activity in smooth muscle cells.	(4)
	High-Fat/High-Glucose Diet	Induces endothelial ARG1 expression, reducing NO bioavailability.	(30)
Infection	Pathogen (e.g., Salmonella)	IL-4 stimulation reprograms L-arginine metabolism to induce ARG1 in macrophages.	(24)
	Fungal Enzyme Homolog (IP_3-4_K)	Cryptococcal ARG1 homolog drives virulence via its inositol polyphosphate kinase activity.	(25)
Broad Mechanisms	Epigenetic (DNA methylation)	Hypomethylation of the ARG1 promoter in MDSCs correlates with high expression.	(18)
	Epigenetic (RNA m6A modification)	ALKBH5-mediated demethylation destabilizes ARG1 mRNA in MDSCs.	(19)
	Microbiome	Altered gut microbiota and L-arginine metabolism influence ARG1 activity in colitis.	(23)
	Pharmacologic Compounds (e.g., CDK8/19 inhibitor)	Enhances p38 MAPK/STAT6 signaling to induce ARG1.	(12)

## 3 Heterogeneous roles of ARG1 in different diseases

This section outlines the fundamental mechanisms of ARG1-polyamine metabolism in key immune cells, providing a basis for understanding its roles in various diseases. The activation and functional regulation of immune cells are closely related to ARG1-polyamine metabolism. This article will analyze the mechanism of ARG1 in T cells, dendritic cells (DCs) and macrophages, and its regulation of immune response.

### 3.1 ARG1-polyamine metabolism in T cell differentiation and dendritic cell function

Polyamine synthesis is one of the most important metabolic changes in the process of T cell activation, and it is a basic process that controls the polarization of CD4^+^ helper T cells (T_H_) into different functional destinies and capacities. Deficiency of ODC, a key enzyme in polyamine synthesis, can markedly cause CD4^+^ T cells to fail to differentiate into the correct subpopulation (31). Deficiency in polyamine-hypusine (a post-translational modification derived from spermidine) drives histone acetylation and tricarboxylic acid (TCA) cycle alterations, causing extensive epigenetic remodeling (31). Beyond its role in lymphocyte biology, ARG1 also functions in innate immune contexts. Following myeloid activation, released extracellular ARG1 localizes to NETs (neutrophil extracellular traps), where it interacts with CTSS (cathepsin S). CTSS then cleaves ARG1, producing major molecular forms with enhanced enzymatic activity at physiological pH (32). The immunomodulatory role of ARG1 extends to DCs, where it interacts with other immunosuppressive pathways. ARG1 and indoleamine 2,3-dioxygenase 1 (IDO1) catalyze L-arginine and L-tryptophan degradation, respectively, leading to local amino acid deprivation (33). Specifically, ARG1 expression and ARG1-dependent polyamines production in DCs activate Src kinase to phosphorylate IDO1 and activate IDO1 signaling to regulate DCs to an IDO1-dependent immunosuppressive phenotype (33).

### 3.2 ARG1-polyamine metabolism in macrophage polarization and efferocytosis

ARG1 is one of the markers of M2 macrophage polarization and plays a central role in mediating key anti-inflammatory and reparative functions. In macrophages, ARG1 and ODC metabolize L-arginine and ornithine derived from apoptotic cells (ACs) to putrescine. This putrescine enhances the stability of HuR-mediated encoded GTP (guanosine triphosphate) exchange factor *Dbl* mRNA, and activates actin to regulate Rac1 to enhance efferocytosis, which prevents necrosis and promotes damage resolution (34).

In pathological contexts, particularly cancer, the activity of myeloid cell-derived ARG1 is a major immunosuppressive mechanism. In pancreatic cancer, ARG1^+^ TAMs drive immunosuppression by depleting L-arginine. Genetic or pharmacological ARG1 inhibition enhances CD8^+^ T cell infiltration and synergizes with anti-PD1 therapy (35, 36). The regulation of ARG1 expression in macrophages involves specific signaling pathways. IL-4 and IL-13 induce cholesterol-25-hydroxylase (Ch25h) expression in tumor-associated macrophages through STAT6, leading to the accumulation of lysosomal 25-hydroxycholesterol (25HC). This metabolite binds to cholesterol-competing G protein-coupled receptor 155 (GPR155) to inhibit kinase mechanistic target of rapamycin complex 1 (mTORC1), resulting in AMPKα activation and STAT6 Ser564 phosphorylation, which ultimately enhances STAT6 activation and ARG1 production (10). However, it has been reported that IL-4 induced ARG1-deficient macrophages significantly enhanced polyamine production, suggesting that there may be a polyamine synthesis pathway independent of ARG1 in macrophages (37).

**Figure 2:**
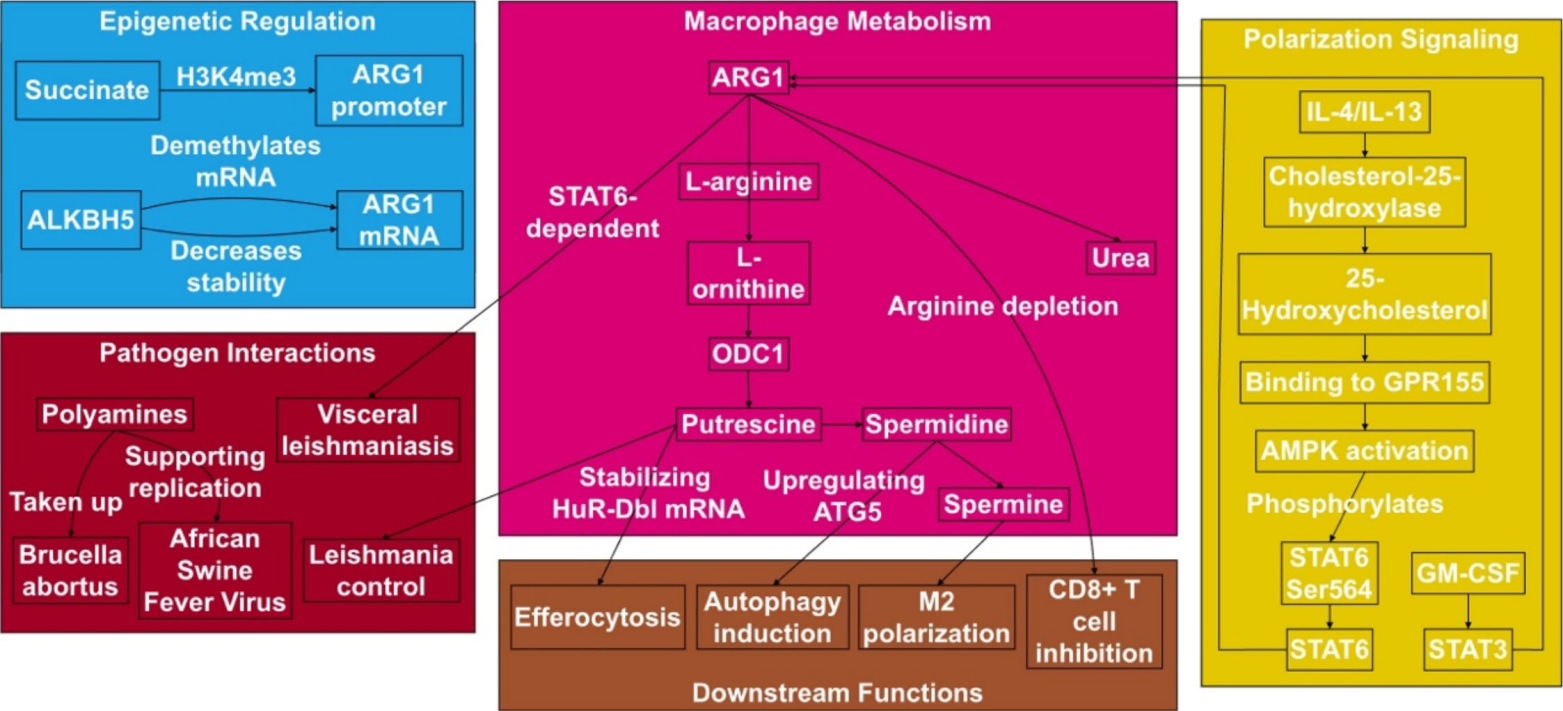
The ARG1-polyamine axis governs macrophage function and host-pathogen crosstalk. Within macrophages, L-arginine is metabolized by ARG1 to L-ornithine, fueling polyamine synthesis via ODC1. ARG1 expression is induced by IL-4/IL-13 (via STAT6) and GM-CSF (via STAT3). Key downstream effects include putrescine-enhanced efferocytosis, polyamine-driven M2 polarization/autophagy, and ARG1-mediated T cell suppression via L-arginine depletion. Pathogens such as *Brucella* and ASFV hijack host polyamines, while putrescine can redirect metabolism to control *Leishmania*. Epigenetic regulators like succinate and ALKBH5 fine-tune ARG1 expression.

The interplay between polyamines, autophagy, and macrophage polarization further modulates inflammatory outcomes. Autophagy can regulate the polarization of macrophage M1/M2 under different inflammatory conditions (38). Polyamines, including putrescine, spermidine, and spermine (SPM), have antioxidant, anti-aging, and autophagy-inducing effects (38). For example, SPM enhances autophagy of hepatic Kupffer cells (KCs) by up-regulating ATG5 expression to inhibit M1 polarization of thioacetamide (TAA)-treated KCs and promote M2 polarization, thereby alleviating liver injury (38). Conversely, inhibition of the ARG1/ODC pathway elevates histone deacetylase 3 (HDAC3), exacerbating IL-1β/TNF-α production. Thus, enhancing ARG1 activity to suppress HDAC3 represents a strategy to attenuate retinal ischemia (3). The regulatory mechanism of ARG1-polyamine metabolism in macrophages and its interaction with other cells and microorganisms are shown in **Figure 2**.

### 3.3 The role of ARG1-polyamine metabolism in tumor

In tumor cells, ARG1-polyamine metabolism plays an important role in tumor growth and drug resistance by influencing cellular metabolic reprogramming and immune microenvironment. The specific mechanism of ARG1-polyamine metabolism in tumor cells and its role in tumorigenesis and development were investigated.

#### 3.3.1 Mechanisms of ARG1-polyamine metabolism in tumor cells

Cancer cells undergo altered, often enhanced, metabolic processes to meet their high biogenetic needs (39). Within this landscape of metabolic reprogramming, the ARG1-polyamine axis plays a crucial role. Cytoplasmic L-ornithine is an intermediate product of the urea cycle and a specific substrate for the production of putrescine by ODC, which is essential for tumor growth (1). Furthermore, L-arginine binding to RBM39 (RNA-binding motif protein 39) mediates upregulation of asparagine synthesis, resulting in increased L-arginine uptake and reduced L-arginine-to-polyamine conversion, leading to high levels of L-arginine accumulation in tumor cells. High levels of L-arginine promote tumor formation by further metabolic reprogramming, namely altering glucose, amino acid, nucleotide, and fatty acid metabolism (40). These studies highlight that ARG1’s oncogenic effects are partly mediated through polyamine-dependent mechanisms.

This metabolic shift toward polyamine synthesis and associated reprogramming is counter regulated by tumor suppressor pathways. As the most commonly mutated gene in human tumors, tumor suppressor p53 inhibits ammonia metabolism by down-regulating CPS1 (carbamoyl phosphate synthase 1), OTC (ornithine transcarbamylase), and ARG1 transcription (39). Conversely, downregulation of these genes activates p53 via MDM2-mediated mechanisms, leading to ammonia accumulation. This reduces translation of *ODC1* mRNA—a rate-limiting enzyme in polyamine biosynthesis—thereby inhibiting polyamine synthesis and cell proliferation (39).

The functional impact of ARG1-polyamine metabolism is evident across different cancer types. In hepatocellular carcinoma (HCC), ARG1 overexpression enhances arginase activity and promotes epithelial-mesenchymal transition (EMT), likely through polyamine-mediated signaling pathways that facilitate metastasis (41). Similarly, in colorectal cancer (CRC), ARG1-driven L-arginine metabolism supports polyamine biosynthesis, which in turn fuels tumor growth and immune evasion (42).

#### 3.3.2 Context-dependent roles of ARG1 in tumor cells and the immune microenvironment

ARG1’s role in cancer is highly context-dependent, influencing both tumor cells and the immune microenvironment. Within tumor cells themselves, ARG1 function exhibits significant variation. The tumor-suppressive function of p53 through inhibiting the urea cycle/polyamine axis can be subverted in cancer. For instance, in CRC, highly expressed lncRNA LVBU (long non-coding RNA regulation via BCL6 (B-cell lymphoma 6 protein)/urea cycle) competitively binds miR-10a/miR-34c to stabilize BCL6. This blocks p53-mediated inhibition of urea cycle/polyamine synthesis genes (ARG1, OTC, ODC1), promoting tumorigenesis (26). ODC1 inhibitors attenuate growth in high-LVBU xenografts (26). Metformin (Met) upregulates AMPK and p53 expression in CRC models, decreases urea cycle enzymes (CPS1, ARG1, OTC, ODC), reduces putrescine levels, and inhibits cancer cell proliferation (1). In the same cancer type, elevated L-arginine levels, driven by low levels of ARG1 β-Hydroxybutyrylation (Kbhb), promote tumorigenicity by inhibiting L-arginine efflux via reduced ARG1-SLC3A2 interaction (42). In contrast, in hepatocellular carcinoma (HCC), ARG1 is significantly downregulated, and its higher expression correlates with more aggressive tumor growth, size, ALT/GGT levels, and poor disease-free survival (DFS) (41). In Huh7 cells, ARG1 overexpression enhances arginase activity, promoting cell viability, migration, invasion, and epithelial−mesenchymal transition (EMT) (41).

Beyond its role in cancer cells, the expression of ARG1 in tumor-associated immune and stromal cells constitutes a key mechanism for fostering an immunosuppressive microenvironment and promoting tumor progression. VISTA (V-domain Ig suppressor of T cell activation) is a key factor in MDSC differentiation. Co-expression of VISTA and ARG1 on tumor-associated myeloid cells correlates with poor survival in endometrial cancer (27). VISTA deletion reduces STAT3-dependent polyamine production, impairing MDSC function (27). ARG1 expression in TAMs is often associated with an M2-like, pro-tumorigenic phenotype (43). However, the cellular source of ARG1 dictates its immunological impact. In non-small cell lung cancer (NSCLC), tumor-associated neutrophils (TANs), not monocytes/macrophages, are a primary source, with Annexin A2 (ANXA2)/Toll-like receptor 2 (TLR2)/myeloid differentiation primary response 88 (MYD88) signaling inducing ARG1 mRNA (44). CAFs in the tumor microenvironment (TME), especially myofibroblasts, use metabolism to reshape the extracellular matrix (ECM) to promote tumor metastasis (15).

The ARG1/iNOS competitive axis for L-arginine metabolism represents a pivotal metabolic switch determining the immune status of the tumor microenvironment. Based on the supporting literature, increased L-arginine availability can indeed promote iNOS expression and activity. For instance, in a study on Mycobacterium tuberculosis infection, L-arginine supplementation was shown to enhance Nos2 expression, which contributed to improved immune defense and tissue repair (45, 46). In the context of cancer, elevated iNOS activity, driven by increased L-arginine, is generally associated with pro-inflammatory, anti-tumorigenic effects. This is because nitric oxide (NO) production can have cytotoxic effects on tumor cells and can promote an immunostimulatory TME. For example, research in lung carcinoma models indicates that restoring L-arginine levels via ARG inhibition can reinvigorate T-cell responses and inhibit tumor growth, a process potentially facilitated by a shift towards iNOS-mediated immunity (47). Conversely, the suppression of iNOS activity, often seen in M2-polarized macrophages, is linked to tumor progression. Therefore, while ARG1 activity depletes L-arginine to inhibit T-cells and promote tumor growth, enhanced L-arginine availability can shift metabolism towards the iNOS pathway, promoting anti-tumor immunity and potentially inhibiting tumor growth.

Recent studies highlight ARG1’s dual role: Myeloid-derived ARG1 promotes immunosuppression, while ARG1 in breast cancer (BC) cells suppresses AKT signaling and correlates with better prognosis (48). ARG1 overexpression in BC reduces xenograft growth (48). In pancreatic ductal adenocarcinoma (PDAC), neutrophil extracellular traps (NETs) sequester CTSS-cleaved ARG1 to suppress CD8^+^ T cells. Targeting NET-ARG1 with antibodies restores T cell function (32).

**Figure 3:**
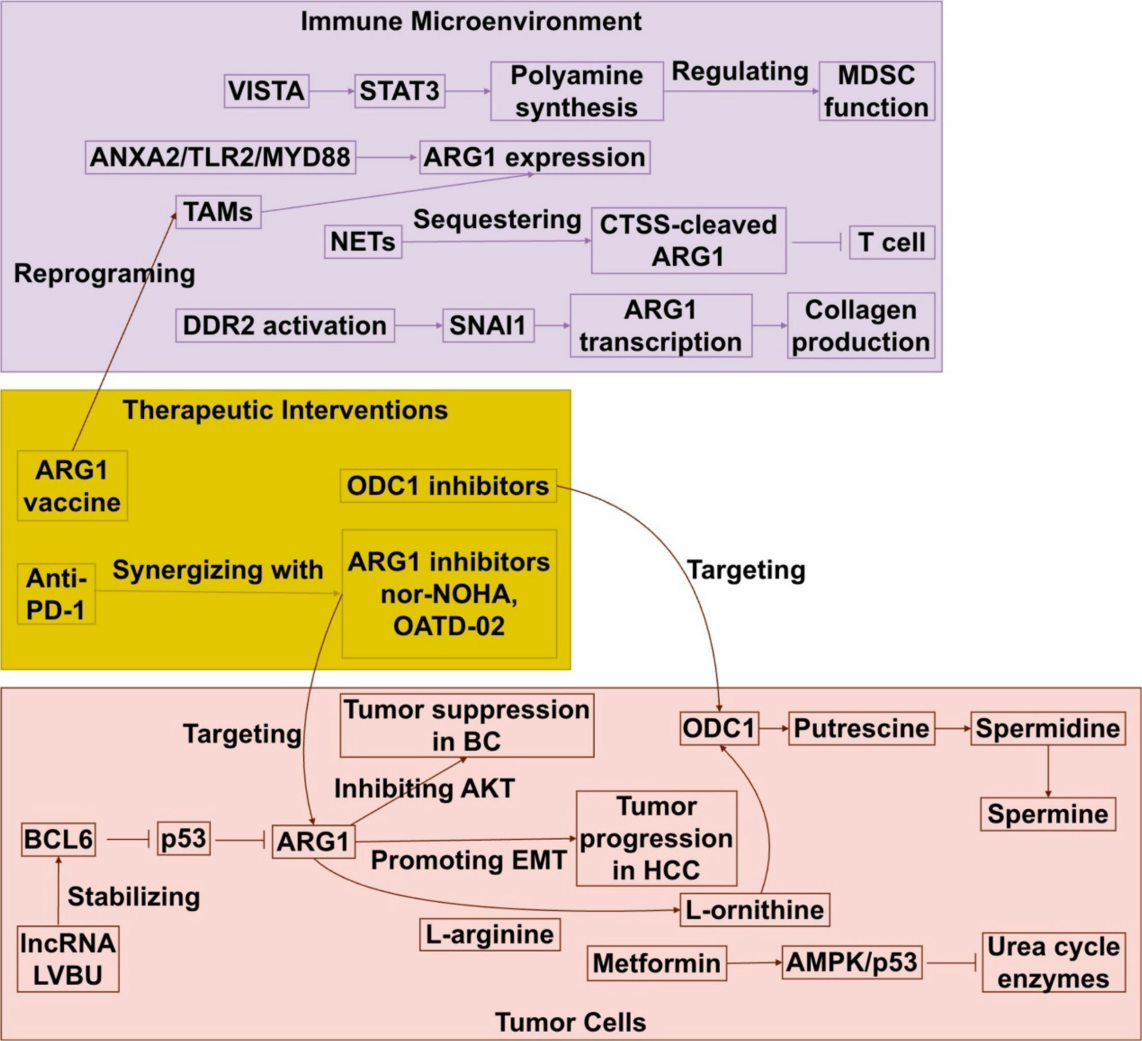
Dual roles and therapeutic targeting of the ARG1-polyamine axis in cancer. In tumor cells, ARG1-driven polyamine synthesis supports growth but is suppressed by p53. The lncRNA LVBU blocks p53 via BCL6, promoting tumorigenesis. ARG1 function is context-dependent, inhibiting (e.g., BC) or promoting (e.g., HCC) cancer. In the TME, ARG1 in myeloid cells (MDSCs, TAMs, TANs) and NETs depletes L-arginine to inhibit CD8^+^ T cells. VISTA-STAT3 signaling enhances polyamine synthesis in MDSCs, while CAFs use DDR2-SNAI1 to upregulate ARG1 and collagen production. Therapeutic strategies include ARG1/ODC1 inhibitors, combination with anti-PD-1, and ARG1 vaccines.

These findings underscore the potential of targeting ARG1-polyamine pathways for cancer therapy. ARG1-based vaccines can induce endogenous antitumor immunity and synergize with checkpoint blockade such as anti-PD-1, thereby increasing T-cell infiltration and shifting the M1/M2 macrophage ratio (49). ARG1 blocking in combination with immune checkpoint inhibitors can restore CD8^+^ T cell function in PDAC tumors *in vitro*, but further exploration is needed (32). As illustrated in **Figure 3**, the ARG1-polyamine axis plays a dual role in cancer by supporting tumor cell proliferation and fostering an immunosuppressive tumor microenvironment, highlighting promising therapeutic targets for intervention.

### 3.4 ARG1 in autoimmune, inflammatory diseases

This section explores the role of ARG1 in autoimmune disorders, and inflammatory conditions, highlighting its context-dependent functions across different diseases. Its role is evident from modulating antiviral responses to driving pathology in chronic inflammatory and fibrotic diseases.

In classic systemic autoimmune diseases, ARG1 often contributes to immunosuppressive or pathogenic networks. In rheumatoid arthritis (RA), myeloid-derived suppressor cells (MDSCs) and TAMs express ARG1, contributing to immune suppression. Triptolide (TPT) treatment reduces MDSC frequency and ARG1 expression, alleviating arthritis (50). The transcription factor c-Jun promotes arthritis by upregulating cyclooxygenase-2 and indirectly inhibiting ARG1 in macrophages (51). In systemic lupus erythematosus (SLE), MDSCs expand and promote T helper 17 (Th17) differentiation in an ARG1-dependent manner. The ARG1/miR-322-5p axis shifts the Th17/T regulatory cell (Treg) ratio, driving disease progression (52, 53). In experimental autoimmune encephalomyelitis (EAE), a model of multiple sclerosis (MS), Astragalus polysaccharide (APS) ameliorates disease by activating the AMPK/JAK/STAT3/ARG1 pathway, inducing MDSCs and increasing ARG1 expression (54). ARG1 is expressed exclusively by infiltrating myeloid cells, not resident microglia, in CNS injury and EAE, suggesting distinct roles for these cell populations (28).

ARG1 is a critical regulator in skin barrier integrity and inflammatory diseases. In psoriasis, ARG1 upregulation in keratinocytes drives polyamine overproduction, which stabilizes self-RNA and promotes dendritic cell activation via Toll-like receptor 7 (TLR7), thereby exacerbating skin inflammation (7). This illustrates how ARG1-mediated polyamine synthesis directly links metabolic reprogramming to autoimmune amplification. IL-17 down-regulates protein phosphatase 6 (PP6) in psoriatic keratinocytes, leading to phosphorylation and activation of the transcription factor CCAAT/enhancer-binding protein beta (C/EBP-β), and subsequent production and accumulation of ARG1. The increased ARG1 activity shifts L-arginine metabolism towards L-ornithine production, which in turn serves as the substrate to drive polyamine synthesis. Polyamines protect self-RNA released by psoriatic keratinocytes from degradation and promote its endocytosis by bone marrow DCs, thereby promoting TLR7-dependent RNA sensing and IL-6 production. Accordingly, ARG1 inhibitors improve skin inflammation in mouse and non-human primate models of psoriasis (7). Abelmoschi Corolla (AC) ameliorates psoriasis by reducing ARG1 expression and L-arginine metabolism in keratinocytes (55). Conversely, in atopic dermatitis (AD), ARG1 is downregulated in keratinocytes, which impairs their differentiation and antimicrobial peptide production. This defect can be rescued by supplementation with downstream polyamines (e.g., putrescine) or urea, underscoring the crucial role of the ARG1-polyamine axis in maintaining skin barrier immunity (6). Furthermore, epidermal ARG1 expression is decreased in delayed healing wounds in humans and mice. Wound repair was significantly delayed and was associated with changes in keratinocyte proliferation, migration, and differentiation in both keratinocyte-specific ARG1 deletion mouse models (K14-cre; Arg1^fl/fl^) and *in vitro* human models using ARG inhibitor (nor-NOHA), revealing the importance of downstream polyamine pathways in repair (56).

The ARG1-polyamine axis is also a key driver in fibrotic processes across different organs. Monocyte-derived macrophages are recruited into injured tissue to induce IL-6 expression in fibroblasts via purinergic receptor P2X purinoceptor 4 (P2rx4) signaling, which in turn induces ARG1 expression in macrophages. ARG1 promotes fibrosis by metabolizing L-arginine to L-ornithine, which fibroblasts use as a substrate to synthesize L-proline, an important component of collagen (57). Complement factor B enhances the activity of the ARG1 enzyme in macrophages, promotes the synthesis of L-ornithine, leads to excessive production of polyamines in macrophages, promotes proliferation of cardiac fibroblasts and collagen production, and leads to progressive cardiac remodeling (CR) (58). By inhibiting ARG1 activity, the inhibitor (N^ω^ -hydroxyl-l-arginine) significantly improved the overall survival rate of uremic mice, inhibited cardiac fibroblast infiltration, and alleviated the progression of CR (58).

ARG1 function extends to other tissue-specific physiological and pathological contexts. Both *ARG1* and *ARG2* mRNA are expressed in human endometrial tissues. However, compared with the proliferative phase of the menstrual cycle, the expression level of ARG2 in human endometrial epithelial cells in the secretory phase is up-regulated, which increases the production of polyamines and L-proline to promote endometrial growth (5). Mouse colon macrophages are located directly near epithelial crypt cells, and the absence of tuberous sclerosis complex 2 (Tsc2) in macrophages can activate mTORC1 signaling and induce polyamine synthesis (59). Epithelial cells take up these polyamines and rewire cell metabolism to optimize proliferation and prevent colitis-induced intestinal damage (59). In bone biology, ARG1 is down-regulated during RANKL-induced differentiation of bone marrow-derived macrophages into osteoclasts and may negatively regulate osteoclast differentiation by promoting NO production (60). The ARG1-polyamine axis also extends its influence to the peripheral nervous system and gut-brain interactions. Enteric-associated neurons (EANs) are closely associated with immune cells and continuously monitor and regulate homeostasis functions in the gut such as movement and nutrient perception (61). In a mouse intestinal infection model, reduced gastrointestinal motility and loss of excitatory intrinsic EANs (iEANs) are mediated by Nlrp6- and casp11-dependent mechanisms (61).

In summary, the expression and functional regulation of ARG1 in immune and non-immune cells have an important influence on immune response and tissue homeostasis across a wide spectrum of diseases.

### 3.5 ARG1 in neurodegenerative diseases and central nervous system injury

This section examines the dual roles of ARG1 in neuroinflammation, neurodegeneration, and central nervous system (CNS) repair, emphasizing its impact on microglia, neurons, and disease outcomes. ARG1 function is highly context-dependent across different neurological conditions.

In acute CNS injury models such as stroke, ARG1^+^ microglia/macrophages are critical for resolving inflammation. Depleting ARG1^+^ cells exacerbates neuronal damage, increases pro-inflammatory cytokines, and impairs functional recovery, highlighting their neuroprotective role (62). Cerebral ischemia causes microglia activation, and the absence of Sema4D promotes the synthesis of polyamines by ARG1 competing with iNOS for L-arginine, which promotes the proliferation of ischemic cortical microglia (63). The supplementation of polyamine putrescine can also promote the proliferation of microglia (63). Activation of the STAT6/ARG1 pathway can promote efferocytosis and regression of inflammation in stroke mouse models (64). In traumatic brain injury models, overexpression of ARG1 in neurons themselves significantly reduced contusion size and index two weeks after controlled cortical injury (2).

In the context of Alzheimer’s disease and amyloid pathology, ARG1 plays a complex role in amyloid-β (Aβ) metabolism. ARG1 haploinsufficiency promotes Aβ deposition and decreases the composition of the regulator-Rag complex involved in mechanistic target of rapamycin complex 1 (mTORC1) signaling and autophagy, exacerbating some behavioral disorders (65). During sustained neuroinflammation driven by IL-1β, centrally derived ARG1^+^ microglia are upregulated and correlate with Aβ plaque clearance, and inducing ARG1^+^ microglia with IL-4 promotes plaque reduction (66). ARG1^+^ microglia are also observed physiologically during development; these resident immune cells are prominently found in regions like the basal forebrain and ventral striatum in early postnatal mice and are rich in phagocytic inclusion bodies (67). Specifically knocking down ARG1 in microglia can lead to loss of cholinergic innervation and impaired maturation of dendritic spines in the hippocampus, further leading to impaired long-term potentiation and cognitive behavioral deficits in female mice (67). ARG is a key enzyme in neurons and glia capable of consuming L-arginine and producing L-ornithine and polyamines, and both inhibition of ARG1 and supplementation of L-arginine impair the phagocytosis of microglia (65).

In tauopathy models such as frontotemporal dementia, gene therapy approaches demonstrate therapeutic potential. Overexpression of ARG1 by adeno-associated virus (AAV) in the CNS of rTg4510 tau transgenic mice decreased the expression of several phosphorylated tau kinases, significantly reduced tau tangling pathology, modulated mammalian target of rapamycin-related proteins to activate autophagy, and alleviated hippocampal atrophy (29).

ARG1 is also implicated in neuropsychiatric conditions and systemic inflammation affecting the brain. In depression, luteolin promotes ARG1^+^ microglial polarization via peroxisome proliferator-activated receptor γ (PPARγ), reducing neuroinflammation and synaptic loss (68). Neuroinflammation is one mechanism by which obesity or a high-fat diet leads to cognitive impairment (69). In models of traumatic brain injury (TBI), skull bone marrow-derived neutrophils (N(skull)) expressing osteocalcin (OCN) and ARG1 infiltrate the brain, where they exhibit immunosuppressive and neuroprotective effects (70).

### 3.6 ARG1 in metabolic diseases, obesity, and vascular disorders

This section discusses the involvement of ARG1 in metabolic syndromes, obesity-related complications, and vascular dysfunction, focusing on endothelial and smooth muscle cells. ARG1 expression in endothelial cells is closely related to a variety of vascular diseases (30, 71–82).

As a key pathogenic factor, ARG1 is increased in expression/activity in endothelial cells (ECs) and is associated with the development of obesity-related type 2 diabetes and related vascular diseases (75). Mechanistically, increased ARG1 activity in vascular endothelial cells contributes to decreased L-arginine and nitric oxide (NO) bioavailability, leading to impaired endothelium-dependent vascular relaxation, arterial fibrosis and hardening, and elevated blood pressure under high-fat and high-sugar diets or in the early course of atherosclerosis in obesity (30, 72). In mice fed with high glucose (25 mM) and high fat (sodium palmitate, 200 μM) or treated with the ARG inhibitor ABH (2-(S)-amino-6-boronohexanoic acid), the expression of tumor necrosis factor-alpha (TNF-α), vascular cell adhesion molecule 1 (VCAM-1), intercellular adhesion molecule 1 (ICAM-1), and monocyte chemoattractant protein-1 (MCP-1) decreased despite weight gain and hyperglycemia (81). ARG1 inhibitors (nor-NOHA) may also help restore dysregulated endothelial cell function by increasing the production of endothelial NOS (eNOS)-dependent NO in endothelial cells (79). Furthermore, in diabetic models, PEG-ARG1 (pegylated ARG1) administered three times a week for two weeks inhibited retinal inflammation (iNOS, IL-1β, TNF-α, IL-6) and elevated markers of oxidative stress in db/db (leptin receptor-deficient) mice, restored blood-retinal barrier (BRB) function, decreased tissue albumin extravasation, and improved endothelial ZO-1 (zonula occludens-1) tight junction integrity (83).

ARG1, positioned at a critical metabolic node, directs L-ornithine derived from the urea cycle into polyamine synthesis and L-proline/collagen production, thereby coordinating cellular proliferation, matrix dynamics, and neuroinflammation. Beyond the endothelium, ARG1 plays a critical and complex role in vascular smooth muscle cells (SMCs) and remodeling. Increased ARG activity provides L-ornithine for the synthesis of polyamines by ODC and L-proline/collagen synthesis by ornithine aminotransferase (OAT) (4). ARG1 inhibits pro-inflammatory cytokines (TNF-α, IL-6) and increases the expression of the anti-inflammatory factor IL-10 by increasing the production of intracellular polyamines, while exogenous L-arginine restores the expression of inflammatory cytokines. Furthermore, ARG1 reduces iNOS activity and is observed to co-localize with iNOS. This process stimulates the proliferation of SMCs in aortic vessels and enhances the stability of atherosclerotic plaques (84). Both mice treated with Ang II (4 weeks) and smooth muscle cells exposed to Ang II (1 μM, 48 h) increased ARG1 and ODC expression/activity, as well as proliferating cell nuclear antigen, hydroxyproline levels, and collagen 1 expression, leading to increased smooth muscle cell proliferation and collagen synthesis. It causes hardening of the aorta, fibrosis and thickening of the aorta and coronary arteries, and ARG1 deletion prevents these changes (4). On the other hand, the absence of ARG1 in red blood cells promotes vascular calcification by enhancing GSNOR (S-nitrosoglutathione reductase) expression and reducing HSP70 (heat shock protein 70)-S-nitrosation, activating NO signaling in smooth muscle cells (85).

At a systemic level, ARG1 activity is elevated in the plasma and exosomes of obese individuals and high-fat diet (HFD)-fed mice, with the liver being a potential source (71, 72). ARG inhibition with nor-NOHA ameliorates obesity-induced hepatic lipid abnormalities and whole-body adiposity by increasing hepatic NO production and activating AMPK (86). Supporting this pathogenic link, elevated plasma ARG1 levels are observed in adolescents with obesity and T2DM, further correlating with endothelial ARG1 expression and the progression of metabolic disease (71, 75). Furthermore, ARG1 polymorphisms are associated with the risk of vascular complications in T2DM patients (87). It is noteworthy that the role of ARG1 extends beyond the vascular system in metabolic contexts. For instance, hematopoietic and endothelial cell deficiency of ARG1, increased availability of L-arginine, and altered gut microbiota and metabolites can accelerate the resolution of colitis (23).

### 3.7 ARG1 in stem cells and regenerative medicine

This section highlights the role of ARG1 in maintaining stem cell quiescence, promoting differentiation, and its implications for liver regeneration and cancer. ARG1-polyamine metabolism plays a key role in the proliferation and differentiation of stem cells. A specific and mechanistic role for ARG1 has been elucidated in liver biology. In hepatic stellate cells (HSCs), ARG1 binds embryonic stem cell-expressed Ras (ERas) through its N-terminal extension (Nex) to maintain cellular quiescence by suppressing mTORC1 activity; disruption of this interaction activates HSCs and promotes fibrosis, linking urea cycle function to liver regeneration (88). The significance of ARG1 in cellular differentiation extends to a clinical oncology context. In non-alcoholic non-viral hepatocellular carcinoma (NANV-HCC), decreased ARG1 correlates with poor differentiation and predicts postoperative recurrence (89). This positions ARG1 as a sentinel for hepatocyte maturity, where its loss marks dedifferentiation and worse clinical outcomes. Furthermore, in polycystic kidney disease (PKD), L-lactic acid produced by cyst-lining epithelial cells (CLECs) up-regulates ARG1 expression in macrophages, increases polyamine synthesis, stimulates CLEC proliferation, and promotes cyst growth (90). Similarly, in the context of osteoarthritis, spermidine (SPD) ameliorates joint pathology by altering macrophage polarization. SPD inhibits macrophage ERK MAPK and p65/NF-κB signaling, promoting a shift from M1 to M2 phenotype. Although not directly protecting chondrocytes, conditioned medium from SPD-treated M1 macrophages inhibits detrimental p38/JNK MAPK signaling in IL-1β-stimulated chondrocytes, thereby promoting chondrocyte anabolism and inhibiting catabolism (91). In summary, ARG1 functions as a critical regulator of stem/progenitor cell state and differentiation, particularly within the liver. The mechanism of action and therapeutic significance of ARG1 across various diseases are summarized in **Table 2**.

**Table 2:** ARG1 in disease contexts: mechanisms and therapeutic implications.

Disease category	Cell type	Pathogenic mechanism	Therapeutic approach	References
Cancer	Myeloid cells	L-arginine depletion → T cell exhaustion	ARG1 inhibitors + anti-PD-1	(27, 32, 35, 47)
	Tumor cells	AKT inhibition (BC) or metabolic reprogramming	ARG1 overexpression (BC context)	(48)
	CAFs	Collagen production → metastasis	DDR2 inhibition	(15)
Inflammatory & Autoimmune Diseases	Keratinocytes	Polyamines → self-RNA sensing → inflammation	ARG inhibitors	(7)
	Macrophages	Altered Th17/Treg ratio (SLE)	ARG1/miR-322-5p targeting	(52, 53)
	Synovial macrophages	M1 polarization → joint destruction	Spermidine supplementation	(91)
Metabolic/ Vascular	Endothelial cells	NO reduction → vasodilation impairment	nor-NOHA, ABH inhibitors	(75, 79, 81)
	Smooth muscle cells	Collagen synthesis → arterial stiffening	ARG1 deletion prevents changes	(4)
Neurodegenerative	Microglia	Inflammation resolution, Aβ clearance	ARG1^+^ cell preservation	(62, 66, 67)
	Neurons	Reduced tau pathology, hippocampal protection	AAV-ARG1 overexpression	(29)
Infection	Macrophages	Polyamine support for intracellular pathogens	Polyamine synthesis inhibition	(92, 93)
		Altered L-arginine metabolism → parasite control	Putrescine supplementation	(94, 95)

## 4 ARG1-polyamine axis in host-microbe interactions and infection pathogenesis

Beyond these direct roles in stem cell biology and human diseases, the ARG1-polyamine axis also serves as a critical interface in host-microbe interactions, influencing the pathogenesis of a wide range of infections, as discussed in the next section. ARG1-polyamine metabolism plays an important role in host-microbial interactions, influencing infection and immune responses. This section discusses its mechanisms of action in bacterial, parasitic, viral, and fungal infections, as well as its connection to the microbiome in disease.

In bacterial infections, the ARG1-polyamine axis can be subverted to promote pathogen persistence. In alternatively activated macrophages (AAMs) induced by IL-4/IL-13, ARG1 expression is upregulated and polyamine synthesis is increased to promote the intracellular survival of Brucella abortus and chronic infection in mice. Inhibition of polyamine synthesis in macrophages, or inactivation of *potIHGF*, the putative transporter encoding putrescine, reduced *Brucella abortus*’ intracellular survival and chronic infection in mice (92). Following *Salmonella* infection, ARG1 expression in macrophages is upregulated. However, deletion or inhibition of ARG1 in macrophages did not affect control of systemic *Salmonella enterica* serovar Typhimurium (S.tm) infection in mice (96). Interestingly, macrophages infected with *S*.tm can be stimulated with IL-4 to reduce bacterial proliferation through metabolic reprogramming of the L-arginine-dependent pathway (24). Therefore, understanding the intracellular nutrient environment that sustains bacterial growth can help in the development of drugs to treat intracellular infectious pathogens.

In the context of enteric infection, muscularis macrophages (MMs) up-regulate neuroprotective programs through β2-adrenergic receptor (β2-AR) signaling and mediate neuronal protection through the ARG1-polyamine axis in response to intracavitary infection (61). Furthermore, in colitis conditions, 3,3’-diindolylmethane (DIM) enhanced macrophage efferocytosis in a metabolically dependent manner through the aryl hydrocarbon receptor (AhR)-Nrf2/ARG1 signal, inhibited the release of pain-causing substance P (SP) and nerve growth factor (NGF), and alleviated visceral pain (97).

The axis also critically influences the outcome of parasitic infections. In Leishmania infections, ARG1 expression in macrophages varies across host species (BALB/c vs. C57BL/6), correlating with disease susceptibility. Putrescine supplementation shifts macrophage metabolism to favor nitric oxide production, reducing parasite burden (95). *L. donovani* infection induces macrophage STAT6 activation and STAT6-dependent ARG1 expression, resulting in progressive visceral leishmaniasis (98). Studies in L. amazonensis infection reveal complex metabolic interplay (95). Compared with L-arginine, spermidine, or SPM supplementation, putrescine decreased the percentage of infected macrophages and ARG1 expression, while increasing transcriptional levels of ARG2, ODC1, spermidine synthase (SPDS), and spermine synthase (SPMS). In contrast, spermidine and SPM supplementation promoted effects opposite to those of putrescine. This study further observed that L-arginine deprivation or putrescine supplementation increased NOS2 expression without enhancing NO production, whereas L-arginine induced NO in uninfected macrophages (94). Following the description of iNOS induction without a corresponding increase in NO production, it is pertinent to consider the mechanisms underlying this dissociation. The observed discrepancy may be attributed to several factors (1): substrate competition, where ARG1−mediated depletion of local L−arginine pools limits iNOS catalytic activity despite its elevated expression (2); iNOS uncoupling due to cofactor deficiency (e.g., BH_4_) or oxidative stress, leading to superoxide rather than NO generation (3); post−translational inactivation of iNOS via S−nitrosylation or protein−protein interactions (4); rapid consumption of NO by reactive oxygen species in the inflammatory milieu; and/or (5) metabolic diversion of L-arginine toward polyamine synthesis, which can modulate iNOS function. Thus, iNOS expression alone does not reliably reflect NO bioavailability; the functional output depends on the integrated metabolic, redox, and regulatory context within the cell. Additionally, L-arginine supplementation during infection decreased IL-1β secretion, while L-arginine alone or combined with putrescine increased MCP-1 expression at 24 hours post-infection (94).

Beyond bacterial and parasitic contexts, ARG1-polyamine metabolism extends to fungal and viral pathogens. In the fungal pathogen *Cryptococcus neoformans*, the ARG1 gene encodes a protein that functions as an inositol polyphosphate kinase (IP_3-4_K) homolog. Its IP_3-4_K catalytic activity drives key virulence traits such as capsule formation and thermotolerance. Inhibiting this catalytic activity disrupts fungal survival, highlighting it as a novel antifungal target (25). Infection of porcine alveolar macrophages with African Swine Fever Virus (ASFV) increases intracellular polyamine levels. Knocking out ARG1 and adding exogenous L-arginine affected ASFV replication, revealing that the virus hijacks host metabolism to promote its own replication (93). In the context of viral infections, ARG1 also regulates adaptive immune responses. During influenza virus infection in mice, CD4^+^ T cell-intrinsic ARG1 expression regulates Th1 response kinetics. ARG1 facilitates the resolution of virus-specific Th1 effectors, promoting effective viral clearance and limiting lung pathology. Conversely, ARG1 deficiency disrupts this process by altering glutamine metabolism; it accelerates Th1 resolution, which improves viral clearance but paradoxically exacerbates lung immunopathology. Rebalancing glutamine flux can normalize the dysregulated Th1 response, highlighting the metabolic control of antiviral immunity by the ARG1-polyamine axis (99). Loss of ARG1 results in altered glutamine metabolism, while rebalancing glutamine flux normalizes cellular Th1 response (99).

The role of the ARG1-polyamine axis is also evident in inflammatory and cancerous contexts involving the microbiome. In CRC, high levels of *Fusobacterium* (particularly *F. animalis*) are associated with increased immune gene expression (e.g., CXCL8, IL-6, SPP1, IDO1) and elevated ARG1 in tumors, although a direct link to M2 macrophage polarization was not significant (100). *Fusobacterium* adhesion protein 2 (Fap2) is constitutively expressed and may influence the pro-inflammatory microenvironment (100). Resveratrol alleviates DSS-induced inflammatory bowel disease (IBD) by regulating the intestinal microbiota-macrophage-L-arginine metabolism axis, increasing ARG1 and decreasing iNOS in macrophages (101). The delineation of ARG1’s multifaceted roles in disease and infection provides a strong rationale for therapeutic targeting, leading to the development of novel inhibitors and intervention strategies. The role of the ARG1-polyamine axis in the interaction between host and microorganisms is summarized in **Table 3**.

**Table 3:** Host-microbe interactions via ARG1-polyamine axis.

Pathogen type	Host cell	Arg1-polyamine role	Outcome	References
Bacterial	Macrophages	Polyamine synthesis → intracellular survival	*Chronic Brucella* infection	(92)
	Macrophages	IL-4 stimulation → metabolic reprogramming	Reduced *Salmonella* proliferation	(24)
	Macrophages	ARG1 hijacking for replication	Enhanced ASFV replication	(93)
Parasitic	Macrophages	STAT6-ARG1 axis activation	Progressive visceral leishmaniasis	(98)
	Macrophages	Putrescine → NOS2 → NO production	Reduced *Leishmania* burden	(94, 95)
Fungal	*C. neoformans*	ARG1 homolog IP_3-4_K activity	Virulence, capsule formation	(25)
Microbiome	Colorectal tissue	*Fusobacterium* association	ARG1 elevation in tumors	(100)
	Intestinal macrophages	Microbiota-L-arginine metabolism axis	IBD resolution with resveratrol	(101)

## 5 Novel ARG1 inhibitors and targeted therapies

Recent structural analyses, including quantitative structure-activity relationship (QSAR) modeling, have identified key molecular features essential for inhibitor binding, such as lipophilic atom proximity to the mass center, donor positioning relative to ring nitrogen, and surface area ratios (9). The enzymatic activity of ARG1 can be modulated by post-translational modifications, including S-nitrosation by nitric oxide (NO) derived from iNOS. This modification requires a direct protein-protein interaction between iNOS and ARG1 (78).

Building on this foundational knowledge, significant progress has been made in developing potent, selective, and clinically relevant ARG1 inhibitors, moving beyond early-generation compounds. Novel small-molecule inhibitors with improved pharmacological properties are now in development. OATD-02 represents a breakthrough as an orally bioavailable, potent dual inhibitor of intracellular ARG1 and ARG2 with a long target residence time, showing monotherapy and combination efficacy in preclinical models and entering clinical evaluation (102). Highly potent third-generation inhibitors, such as NED-3238, exhibit IC50 values in the low nanomolar range (1.3 nM for ARG1), representing a >1000-fold increase in potency over earlier inhibitors like ABH and nor-NOHA (103). Novel chemotypes, including boronic acid-based piperidine derivatives, offer promising extracellular ARG inhibition (IC50 up to 160 nM) with favorable pharmacokinetics (104). Natural product-derived inhibitors (e.g., THSG, TDF) provide non-competitive inhibition and demonstrate efficacy in improving endothelial function in preclinical models (76, 77).

Beyond traditional small molecules, innovative delivery and targeting strategies are enhancing therapeutic potential and specificity. Liposomal encapsulation of nor-NOHA tunably modulates release kinetics, addressing rapid clearance and improving pharmacokinetics (105). Engineered mesenchymal stem cell-derived extracellular vesicles (nor@MSC-EVs) have been designed to scavenge self-antigens and modulate ARG1/polyamine signaling in DCs, showing promise for autoimmune disease therapy such as psoriasis (106). ARG1-targeted vaccines can reprogram tumor-associated macrophages and synergize with anti-PD-1 checkpoint blockade, inducing endogenous antitumor immunity (107). Furthermore, allosteric inhibition strategies are emerging; targeting the ARG1 trimerization interface offers a novel approach for selective enzymatic blockade (108).

The therapeutic application of targeting this axis is increasingly sophisticated, guided by biomarker development and rational combination strategies. Pharmacological or genetic inhibition of ARG1 not only restores L-arginine availability but also downregulates polyamine synthesis, offering a dual therapeutic strategy. For example, in psoriasis, Abelmoschi Corolla extract reduces ARG1 expression and polyamine production, alleviating skin lesions (55). In glioblastoma, ARG1 inhibitors alter the immunosuppressive microenvironment and reduce polyamine-driven tumor growth (109). These approaches highlight the potential of targeting the ARG1-polyamine axis to simultaneously modulate immune and proliferative pathways.

**Figure 4:**
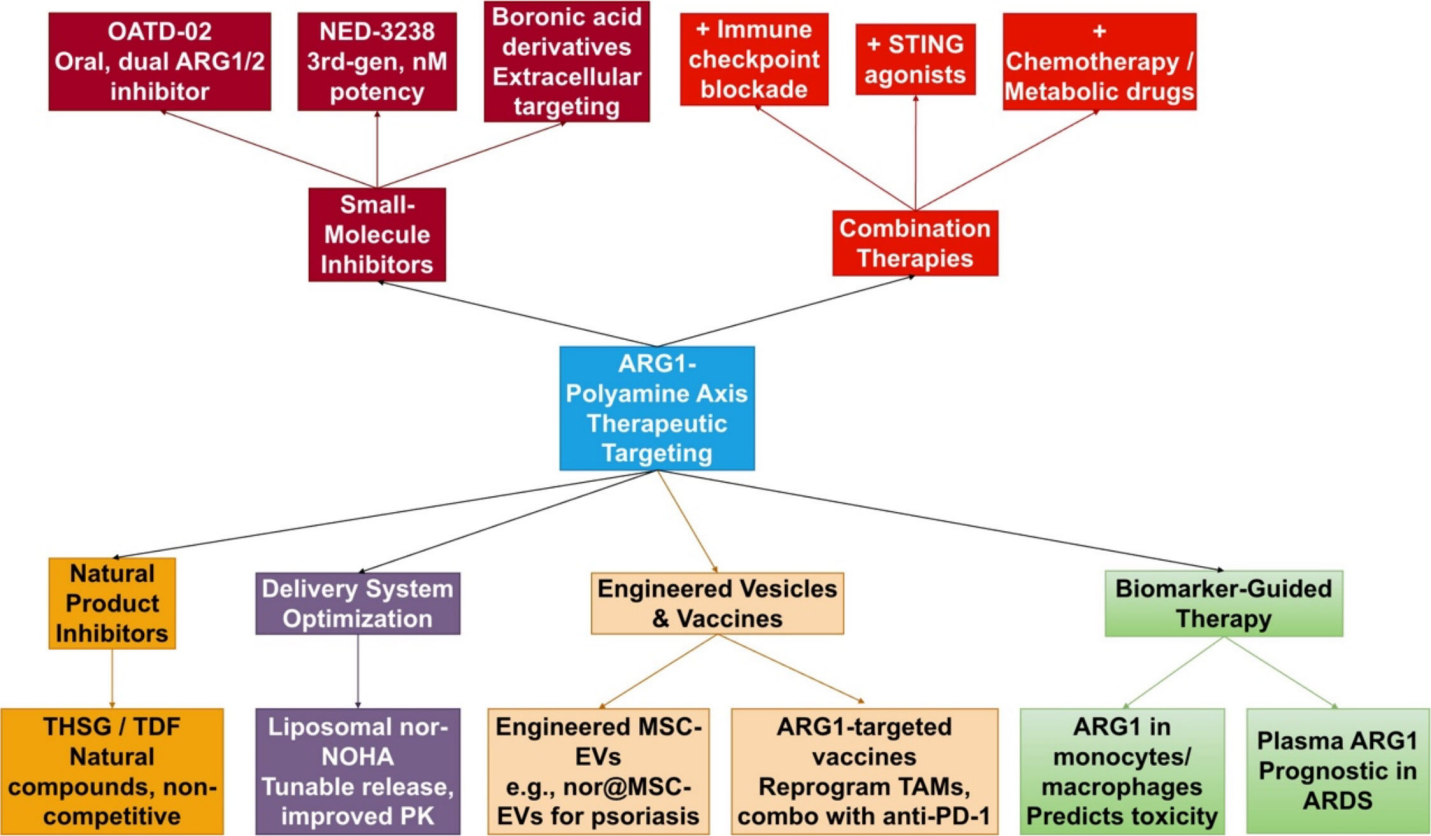
Novel therapeutic strategies targeting the ARG1-polyamine axis. Emerging approaches include next-generation small-molecule inhibitors (e.g., OATD-02, NED-3238), natural product-derived inhibitors, advanced delivery systems (liposomal encapsulation, engineered extracellular vesicles), ARG1-targeted vaccines, and combination therapies with immunotherapy. Biomarker development facilitates patient stratification. These strategies aim to overcome limitations of earlier inhibitors and achieve precise modulation of the ARG1-polyamine axis in cancer, inflammatory, and metabolic diseases.

Therapeutic application is increasingly guided by biomarker development. ARG1 expression in monocytes-derived macrophages predicts radiation-induced skin toxicity in breast cancer patients (110), and plasma ARG1 levels show prognostic value in sepsis-induced acute respiratory distress syndrome (ARDS) (111), supporting patient stratification. Combination therapies are a major focus, with ARG1 inhibition demonstrating significant synergy with immune checkpoint blockade (e.g., anti-PD-1) and STING agonists in poorly immunogenic tumors (47). As summarized in **Figure 4**, these advances highlight a diversified and evolving therapeutic landscape, moving from broad ARG inhibition towards cell-specific targeting, enhanced drug delivery, and rational combination strategies.

## 6 Conclusions and future perspectives

In summary, ARG1-polyamine metabolism has different functions and regulatory mechanisms in multiple cell types, and its role in disease occurrence and progression provides a new theoretical basis for precision medicine and targeted therapy. This axis acts as a double-edged sword in health and disease, demonstrating remarkable cell-type and context-dependent functional diversity. For instance, in the TME, myeloid-derived ARG1 is largely immunosuppressive, while its role in certain tumor cells can be tumor-suppressive. In neurodegenerative diseases, ARG1^+^ microglia/macrophages can be protective by resolving inflammation and clearing debris, whereas in metabolic diseases, endothelial ARG1 drives vascular dysfunction.

Given this complexity, cell-specific targeting strategies are essential. Further study of the cell-specific mechanism of ARG1-polyamine metabolism is expected to provide new strategies and targets for the treatment of cancer, cardiovascular diseases, neurodegenerative diseases and other diseases. Emerging therapeutic strategies include the development of ARG1 inhibitors, such as allosteric ARG1 inhibitors that disrupt trimerization and offer selective enzymatic blockade, overcoming challenges posed by the catalytic site’s polarity (108). Other approaches comprise ARG1-targeted vaccines that reprogram TAMs and enhance checkpoint inhibitor efficacy (107), as well as engineered extracellular vesicles (e.g., nor@MSC-EVs) that inhibit ARG1/polyamine signaling in DCs to alleviate psoriasis (106). Regarding microbiome modulation, probiotics (e.g., *Lactobacillus*) reduce luminal polyamines and synergize with ARG1 blockade in colitis treatment (23). The ongoing clinical development of agents like OATD-02 heralds a new era of ARG-targeted therapy.

Future studies should further dissect the cell-type-specific contributions of ARG1-derived polyamines in disease contexts. For instance, in neurodegenerative conditions like Alzheimer’s disease, ARG1 in myeloid cells influences amyloidosis and autophagy via polyamine-sensitive pathways (65). Similarly, in metabolic disorders, ARG1 in endothelial cells contributes to vascular dysfunction through polyamine-mediated oxidative stress (30). Targeting the ARG1-polyamine axis with selective inhibitors or polyamine antagonists may thus offer novel combinatorial treatment strategies across a spectrum of diseases.

To fully realize this therapeutic potential, future research should focus on several key areas. These include: 1) Delineating the precise molecular switches that determine ARG1’s pro- vs. anti-tumor, pro- vs. anti-inflammatory roles; 2) Developing highly specific inhibitors, allosteric modulators, and targeted delivery systems (e.g., nanoparticles, engineered vesicles) to manipulate ARG1 activity in desired cell populations; 3) Exploring combination therapies that integrate ARG1 blockade with immunotherapy, chemotherapy, or metabolic drugs; 4) Validating ARG1 as a predictive biomarker in larger clinical cohorts across different diseases.

## Glossary

AAV: Adeno-associated virus
ABH: 2-(S)-amino-6-boronohexanoic acid
AC: Abelmoschi Corolla
AD: Atopic dermatitis
AHR: Aryl hydrocarbon receptor
ALKBH5: AlkB homolog 5
AMPKα: AMP-activated protein kinase α
ANXA2: Annexin A2
APS: Astragalus polysaccharide
ARG1: Arginase 1
ASFV: African Swine Fever Virus
ATG5: Autophagy related 5
BC: Breast Cancer
BCL6: B-cell lymphoma 6 protein
BRB: Blood-retinal barrier
CAFs: Cancer-associated fibroblasts
C/EBP-β: CCAAT/enhancer-binding protein beta
Ch25h: Cholesterol-25-hydroxylase
CLECs: Cyst-lining epithelial cells
CM: Conditioned medium
CNS: Central nervous system
CRC: Colorectal Cancer
CR: Cardiac remodelling
CTSS: Cathepsin S
DDR2: Discoidin Domain Receptor 2
DFS: Disease-free survival
DIM: 3,3’-Diindolylmethane
DNMT3A: DNA methyltransferase 3 alpha
EAE: Experimental autoimmune encephalomyelitis
EANs: Enteric-Associated Neurons
ECM: Extracellular matrix
EMT: Epithelial-mesenchymal transition
ERas: Embryonic stem cell-expressed Ras
Fap2: Fusobacterium adhesion protein 2
GM-CSF: Granulocyte-macrophage colony-stimulating factor
GPR155: G protein-coupled receptor 155
GSNOR: S-nitrosoglutathione reductase
HCC: Hepatocellular Carcinoma
HDAC3: Histone deacetylase 3
HDAC4: Histone deacetylase 4
HFD: High-fat diet
HSCs: Hepatic stellate cells
HSP70: Heat shock protein 70
IBD: Inflammatory bowel disease
ICAM-1: Intercellular Adhesion Molecule 1
IDO1: Indoleamine 2,3-dioxygenase 1
iEANs: Intrinsic enteric-associated neurons
IL-1β: Interleukin-1 beta
IL-4: Interleukin-4
IL-6: Interleukin-6
IL-10: Interleukin-10
IL-13: Interleukin-13
IP_3-4_K: Inositol polyphosphate 3–4 kinase
iNOS: Inducible nitric oxide synthase
LVBU: Long non-coding RNA Regulation Via BCL6/Urea Cycle
MAPK: Mitogen-activated protein kinase
MCP-1: Monocyte chemoattractant protein-1
MDSC: Myeloid-Derived Suppressor Cells
Met: Metformin
m6A: N6-methyladenosine
MMs: Muscularis macrophages
mTORC1: Mechanistic target of rapamycin complex 1
MYD88: Myeloid differentiation primary response 88
NANV-HCC: Non-alcoholic non-viral hepatocellular carcinoma
NETs: Neutrophil Extracellular Traps
Nex: N-terminal extension
NGF: Nerve growth factor
NO: Nitric Oxide
nor-NOHA: N(ω)-hydroxy-nor-L-arginine
NOS: Nitric Oxide Synthase
Nrf2: Nuclear factor erythroid 2-related factor 2
NSCLC: Non-small cell lung cancer
OAT: Ornithine aminotransferase
OCN: Osteocalcin
ODC: Ornithine Decarboxylase
OTC: Ornithine Transcarbamylase
OXCT1: 3-Oxoacid CoA-Transferase 1
P5C: Pyrroline-5-carboxylate
PDAC: Pancreatic Ductal Adenocarcinoma
PEG-ARG1: Pegylated ARG1
PKD: Polycystic kidney disease
PP6: Protein phosphatase 6
PPARγ: Peroxisome proliferator-activated receptor gamma
RA: Rheumatoid arthritis
RBM39: RNA-binding motif protein 39
SLE: Systemic lupus erythematosus
SMC: Smooth muscle cell
SP: Substance P
SPD: Spermidine
SPM: Spermine
STAT3: Signal transducer and activator of transcription 3
STAT6: Signal transducer and activator of transcription 6
T2DM: Type 2 diabetes mellitus
TAA: Thioacetamide
TAMs: Tumor-Associated Macrophages
TANs: Tumor-associated neutrophils
TBI: Traumatic brain injury
TCA: Tricarboxylic acid
TET2: Tet methylcytosine dioxygenase 2
Th17: T helper 17 cell
TLR2: Toll-like receptor 2
TLR7: Toll-like receptor 7
TME: Tumor microenvironment
TNF-α: Tumor necrosis factor-alpha
TPT: Triptolide
Treg: Regulatory T cell
Tsc2: Tuberous sclerosis complex 2
UC: Urea Cycle
VCAM-1: Vascular cell adhesion molecule 1
VEGFRI: Vascular endothelial growth factor receptor 1
VISTA: V-domain Ig suppressor of T cell activation
25HC: 25-hydroxycholesterol
β2-AR: β2-adrenergic receptor
